# Knowledge, attitude, and practice of intensive care nurses toward delirium: a multicenter cross-section study

**DOI:** 10.3389/fmed.2025.1552923

**Published:** 2025-09-10

**Authors:** Dabing Dai, Yongming Tian, Wenwen Jing, Huan Liu, Yu Xu

**Affiliations:** Department of Critical Care Medicine, West China Hospital of Sichuan University, Chengdu, China

**Keywords:** intensive care unit, cross-sectional, attitude, knowledge, practice, delirium

## Abstract

**Background:**

Delirium is a common and serious complication among patients in the Intensive Care Unit (ICU). Nurses have an essential role in dealing with delirium in critical illness. However, understanding of ICU nurses’ knowledge, attitude, and practice (KAP)on delirium is limited, and large-scale survey lacking.

**Objectives:**

To explore the status and related factors of KAP of delirium among critical care nurses in China.

**Methods:**

From February to May 2019, a cross-sectional survey was conducted among 3,199 intensive care nurses from 160 hospitals across China using a convenience sampling approach. An online questionnaire was designed and distributed to evaluate the KAP of nurses. Univariate and multivariate linear regression analyses were used to assess the factors that influenced ICU nurses’ KAP.

**Results:**

A total of 3,199 valid questionnaires were collected. Overall, the scores of ICU nurses’ knowledge, attitude, and practice regarding delirium assessment were 38.00 (33.00, 44.00), 23.00 (19.00, 26.00), and 16.00 (12.00, 19.00), respectively. Among the respondents, the proportions of individuals providing positive responses regarding their knowledge, attitude, and practice scores were 48.59%, 58.27%, and 40.52%, respectively. The attitude scores of the nurses were significantly related to knowledge scores (*p* < 0.001). Moreover, the scores for nurses’ practice show a significant correlation with their knowledge scores (*p* < 0.001) and attitudes scores (*p* < 0.001). Multivariate linear regression analysis showed a higher level of education (P = 0.037), surgical ICU (*P* = 0.02), private hospital (*P* < 0.001), clinical position (*P* = 0.007), shorter working hours (*P* < 0.001), primary professional title (*P* < 0.001), and previous experience in delirium nursing (*P* < 0.001)and training (*P* < 0.001)were related to higher practice scores.

**Conclusion:**

The knowledge of delirium among ICU nurses is at a moderate level. While these nurses exhibit a positive attitude toward this issue, their actual practices tend to reflect a relatively negative approach. Managers and nursing researchers should develop and offer standardized training for critical care nurses to improve their KAP toward delirium in critically ill patients.

## 1 Introduction

Delirium is a form of acute cognitive impairment marked by sudden mental confusion, difficulty focusing, and altered states of awareness (1, 2). As the most prevalent neuropsychiatric condition in intensive care units, it affects nearly 70% of critically ill patients (2). The onset of delirium dramatically raises the likelihood of death, extends hospital stays, drives up medical expenses, and heightens the risk of developing dementia later in life (3, 4). For these reasons, taking proactive steps—such as timely evaluation, detection, prevention, and intervention—can enhance ICU patient outcomes and make more efficient use of healthcare resources. Existing clinical guidelines emphasize the importance of using standardized diagnostic tools for early delirium identification. Commonly employed assessment methods include the Sedation-Agitation Scale (SAS), the Confusion Assessment Method for the ICU (CAM-ICU), the Intensive Care Delirium Screening Checklist (ICDSC), and the Nursing Delirium Screening Scale (Nu-DESC) (5–8). ICU nurses are instrumental in conducting these evaluations and screenings (9). A global survey of 1,521 ICU physicians revealed that approximately 70% of ICUs had incorporated routine delirium assessments into their protocols. Despite widespread recognition among ICU professionals that delirium is both prevalent and clinically significant, only 42% of units actually employ validated assessment tools for evaluation (10). Implementation continues to face multiple obstacles, including insufficient provider awareness, skepticism toward screening instruments, and a poor grasp of delirium’s clinical impact (11). Compounding these issues, some practitioners view formal delirium assessment as unnecessarily disruptive to workflow, creating practical challenges in patient care settings (12–15).

Despite having a solid theoretical grounding in delirium, ICU nurses in clinical settings frequently encounter obstacles in assessment, resulting in gaps in early detection and effective management of the condition. Current delirium care tends to prioritize patients’ physical health, often overlooking critical evaluations of cognitive function (16). This oversight complicates timely and appropriate delirium assessment and intervention (17–19). Consequently, examining ICU nurses’ KAP concerning delirium is essential for improving patient outcomes. While existing research has explored the role of knowledge in delirium care, few studies have investigated attitudes and practical application (11, 20). A recent study by Zhou et al. (21) analyzed KAP related to delirium and its subtypes among ICU nurses, finding that while Chinese nurses held favorable attitudes, their knowledge and practical skills required further development. However, the study’s limited sample size and narrow scope of influencing factors may have introduced bias (21). To address these gaps, our study, guided by the KAP model, employed a nationwide cross-sectional survey using self-reported questionnaires. This approach allowed us to evaluate delirium-related KAP among critical care nurses across China and identify key contributing factors (22).

To the best of our knowledge, this study represents the most extensive multi-center cross-sectional survey ever undertaken across mainland China. Breaking new ground, it zeroes in on ICU nurses from hospitals of all tiers—a demographic previously overlooked—to examine real-world challenges like resource constraints, training gaps, and variations in patient profiles, while categorizing participants through a culturally relevant framework unique to China. What truly sets this research apart is its holistic approach. Earlier studies typically examined just one aspect—be it knowledge or attitudes—whereas this investigation methodically synthesizes the critical triad of KAP. It goes beyond surface-level analysis to explore pressing concerns: the nuances of non-pharmacological interventions, the imperative of delirium screening, how assessment tools are utilized in practice, and whether nurses have undergone formal delirium training. These deliberate methodological innovations allow the study to paint a vivid, up-to-date picture of how ICU nurses perceive, approach, and handle delirium care in the wake of current clinical guidelines. The findings offer invaluable foundational insights, paving the way for tailored, context-sensitive strategies—whether through targeted training programs, streamlined workflows, or enhanced institutional support—to elevate delirium management nationwide.

## 2 Materials and methods

### 2.1 Study design and population

This research was a multi-center, cross-sectional investigation conducted through convenience sampling, targeting ICU nurses across 160 hospitals situated in 22 provinces and three municipalities directly governed by the central authorities in China. The data collection took place between February and May of 2019. For a detailed breakdown of the participants’ geographic distribution, please refer to Supplementary Figure 1. The study adhered to the guidelines outlined by the STROBE statement for observational research in epidemiology (23). Eligibility criteria included: (1) holding a registered nurse license, (2) having at least one year of ICU experience and actively practicing in the unit, (3) being a nursing manager involved in clinical duties, and (4) voluntarily agreeing to participate. Participants were excluded if they: (1) were absent due to reasons such as sick leave, personal leave, or training commitments, (2) were nursing students, interns, or trainees, (3) chose not to participate, or (4) mainly dedicated their time to teaching or scientific research instead of clinical practice.

### 2.2 Ethical approval

Before conducting this research, ethical approval was obtained from the Ethics Committee of West China Hospital, Sichuan University (Number: 2019608). All participants received comprehensive documentation outlining the research objectives through email correspondence, enabling them to make informed decisions about their voluntary involvement. We explicitly emphasized their right to withdraw from the study without penalty at any stage. During the entire investigation, we maintained rigorous standards to protect participants’ anonymity, ensure data confidentiality, and uphold principles of equitable treatment throughout the research process.

### 2.3 Data collection

Between February and May of 2019, we gathered data through a carefully designed questionnaire informed by existing literature. Participants received digital consent forms and surveys distributed via the “Questionnaire Star” platform. To maintain data integrity, we made every question compulsory and restricted submissions to one per IP address. Additionally, we also excluded the responses that were submitted within 100 s, the responses from non-mobile devices, and the data with errors, in order to enhance the reliability and validity of the research results.

### 2.4 Questionnaire

Drawing upon the principles of the KAP framework, a panel of seven medical specialists—including an intensivist, a neurologist, a psychiatrist, two nursing administrators, and two practicing nurses—collaborated to design a survey instrument after a thorough literature review. The questionnaire was then field-tested with 262 clinical nurses and nursing supervisors to assess its psychometric properties. Reliability analysis yielded an excellent overall Cronbach’s alpha of 0.908, with subscale coefficients of 0.897 (knowledge), 0.806 (attitude), and 0.747 (practice), all surpassing the 0.7 threshold for acceptable internal consistency (24). Construct validity was assessed using the Kaiser-Meyer-Olkin (KMO) measure, and a KMO value of 0.927 was obtained, indicating good sampling adequacy for factor analysis (24). To establish construct validity, researchers performed confirmatory factor analysis (CFA) using Amos 24.0, examining both model fit (Supplementary Table 1) and convergent validity (Supplementary Table 2). The findings confirmed the instrument’s robust validity across all measured dimensions.

The survey instrument was divided into two main sections comprising 37 questions in total: a 13-item demographic profile and a 22-item KAP assessment. The socio-demographic section includes: hospital level, hospital nature, hospital type, department, age, gender, education, professional title, service years, ICU work duration, position, previous delirium nursing experience and previous delirium knowledge training. The KAP section is constructed based on prior research (12, 15, 25–27). The KAP section includes: knowledge (11 items), attitude (6 items), and practice (5 items). All projects are evaluated using a five-point Likert scale (28). The knowledge dimension mainly reflects an individual’s objective cognitive level regarding knowledge related to delirium, covering their basic understanding of delirium, preventive measures, and the awareness of related risks. The options are “not at all familiar,” “somewhat familiar,” “familiar,” “mostly familiar,” and “mastered,” with scores from 1 to 5. The total score ranges from 11 to 55, where a higher score indicates better knowledge of delirium. The attitude dimension reflects an individual’s tendency in evaluating the assessment of delirium. It specifically includes aspects such as the cognition of perceived benefits and obstacles, emotional responses, and behavioral intentions. Options include “Strongly Disagree,” “Disagree,” “Neutral,” “Agree,” and “Strongly Agree,” with scores from 1 to 5. The total attitude score ranges from 6 to 30, where a higher score reflects a more positive attitude toward delirium. The practical dimension reflects an individual’s performance in actual work regarding the assessment of delirium. It specifically involves aspects such as the frequency of behavior occurrence, the standardization of operations, and the continuity of practice. The options are “Never,” “Occasionally,” “Sometimes,” “Often,” and “Always,” corresponding to scores of 1 to 5. The total score ranges from 5 to 25, with higher scores indicating more active delirium practice.

### 2.5 Sample size


$$n=\left(Z\,\_\,{\left(1-\alpha /2\right)}^{\wedge }\,2^{*}\,p^{*}\,\left(1-p\right)\right)/E^{\wedge }\,2$$

In this formula, n is the required sample size, Z_1–α/2_ is the standard normal variate corresponding to a 95% confidence level (1.96), p is the expected proportion (set as 0.5 to maximize sample size), and E is the allowable margin of error (set at 0.05). Based on this calculation, the minimum sample size required was 384. To account for an estimated 80% response rate, the final target sample size was adjusted to 482.

### 2.6 Statistical analysis

All statistical analyses were conducted using IBM SPSS Statistics, Version 26.0 (IBM Corp., Armonk, NY). Categorical data were summarized as frequencies (*n*) with corresponding percentages (%). For the purposes of this study, responses scoring 4 or 5 on individual items were categorized as positive (29). First, the normal distribution test is performed, and the score distribution of each dimension does not fit the normal distribution. Continuous variables were described using median (IQR). Non-parametric tests—either the Mann-Whitney U test or the Kruskal-Wallis test—were employed for continuous variables that deviated from normality. To examine associations between knowledge, attitude, and practice in delirium assessment, Pearson’s correlation coefficients were computed. In the univariate analysis, variables with statistical significance were selected, and after excluding the variables with a correlation coefficient greater than 0.7 through Spearman correlation analysis, a multiple linear regression model was constructed. Stepwise regression was applied, with inclusion and exclusion thresholds set at *p* < 0.05 and *p* > 0.10, respectively.

## 3 Results

The research distributed 3,300 surveys, refining the total to 3,199 usable responses post the removal of 101 unusable entries, yielding a 96.9% valid response rate.

### 3.1 KAP scores by sample characteristics

The study included 3,199 participants, predominantly frontline clinical nurses who made up 91.9% of the sample. Women accounted for the overwhelming majority at 90.72%. Nearly half of the respondents worked in tertiary hospitals, with most falling between 30 and 40 years of age. A bachelor’s degree was the most common educational background (60.27%), while general ICU nurses represented 73.87% of the cohort. In terms of professional ranking, primary titles were held by 83.71% of participants. Additionally, a significant portion had prior experience caring for delirium patients (88.53%), and the vast majority (83.81%) had undergone formal delirium training. Furthermore, comparisons of knowledge, attitude, and practice scores by sample characteristics consistently showed significant differences in the following variables: hospital levels, hospital types, professional titles, position, service years, previous delirium nursing experience, and previous delirium knowledge training (*P* < 0.05; Table 1)

**TABLE 1:** Demographic characteristics and knowledge, attitude, and practice (KAP) scores.

Characteristic	*N* (%)	Knowledge	Attitude	Practice
**Hospital level**				
Tertiary hospital	1,553 (48.55)	37.00 (33.00, 43.00)	22.00 (19.00, 25.00)	16.00 (11.00, 19.00)
Secondary hospital	756 (23.63)	38.00 (33.00, 44.00)	23.00 (19.00, 26.00)	16.00 (12.00, 19.00)
First-level hospital	890 (27.82)	38.00 (33.00, 45.00)	23.00 (20.00, 26.00)	16.00 (12.00, 19.00)
χ^2^	–	19.49*	15.46*	9.07*
*P*	–	< 0.001	< 0.001	0.011
**Hospital nature**				
State hospital	2,568 (80.28)	37.00 (33.00, 43.00)	22.00 (19.00, 25.00)	15.00 (11.00, 18.00)
Private hospital	631 (19.72)	40.00 (34.00, 47.00)	24.00 (21.00, 27.00)	17.00 (13.00, 20.00)
Z	–	−7.24	−8.52	−7.99
*P*	–	< 0.001	< 0.001	< 0.001
**Hospital type**				
General hospital	2,840 (88.78)	38.00 (33.00, 44.00)	23.00 (19.00, 26.00)	16.00 (12.00, 19.00)
Special hospital	359 (11.22)	38.00 (33.00, 43.00)	22.00 (20.00, 26.00)	15.00 (11.00, 18.00)
Z	–	−0.59	−0.35	−2.89
*P*	–	0.553	0.727	0.004
**Department**				
Medical ICU	352 (11.00)	38.00 (33.00, 44.00)	23.00 (20.00, 26.00)	16.00 (12.00, 19.00)
Surgical ICU	484 (15.13)	38.00 (33.00, 44.00)	22.00 (18.00, 25.00)	16.50 (13.00, 19.00)
General ICU	2363 (73.87)	38.00 (33.00, 44.00)	23.00 (19.00, 25.00)	16.00 (11.00, 19.00)
χ^2^	–	1.85*	11.11*	10.55*
*P*	–	0.397	0.004	0.005
**Age (years)**				
< 30	1,817 (56.80)	37.00 (33.00, 44.00)	22.00 (19.00, 25.00)	16.00 (11.00, 19.00)
30–40	1,351 (42.23)	38.00 (33.00, 44.00)	23.00 (19.00, 26.00)	16.00 (12.00, 19.00)
>40	31 (0.97)	36.00 (33.00, 43.50)	22.00 (19.50, 25.00)	14.00 (11.00, 19.50)
χ^2^	–	5.79*	5.17*	11.37*
*P*	–	0.055	0.075	0.003
**Gender**				
Male	297 (9.28)	38.00 (33.00, 44.00)	22.00 (19.00, 26.00)	16.00 (12.00, 19.00)
Female	2,902 (90.72)	38.00 (33.00, 44.00)	23.00 (19.00, 26.00)	16.00 (12.00, 19.00)
Z	–	−0.12	−0.06	−1.72
*P*	–	0.905	0.949	0.086
**Education**				
Associate degree and below	1,231 (38.48)	37.00 (33.00, 44.00)	22.00 (18.00, 25.00)	16.00 (12.00, 19.00)
Bachelor’s degree	1,928 (60.27)	38.00 (33.00, 44.00)	23.00 (20.00, 26.00)	16.00 (11.00, 19.00)
Master’s degree and above	40 (1.25)	42.50 (32.75, 45.00)	24.50 (22.50, 28.00)	18.00 (14.75, 19.00)
χ^2^	–	3.24*	47.45*	8.06*
*P*	–	0.197	< 0.001	0.018
**Professional title**				
Primary	2,678 (83.71)	38.00 (33.00, 44.00)	22.00 (19.00, 25.00)	16.00 (12.00, 19.00)
Intermediate	434 (13.57)	37.00 (32.00, 44.00)	23.00 (20.00, 26.00)	14.00 (9.00, 18.00)
Senior	87 (2.72)	42.00 (37.00, 46.50)	25.00 (23.00, 28.00)	16.00 (12.00, 18.00)
χ^2^	–	15.44*	45.29*	50.62*
**Hospital level**				
*P*	–	< 0.001	< 0.001	< 0.001
**Service years**				
< 5	1,301 (40.67)	37.00 (32.00, 43.00)	22.00 (18.00, 25.00)	16.00 (13.00, 19.00)
5–10	911 (28.48)	38.00 (33.00, 44.00)	22.00 (19.00, 26.00)	16.00 (12.00, 19.00)
> 10	987 (30.85)	39.00 (33.00, 45.00)	23.00 (20.00, 26.00)	15.00 (10.00, 18.00)
χ^2^	–	31.92*	54.65*	28.50*
P	–	< 0.001	< 0.001	< 0.001
**ICU Work Duration(years)**				
< 5	1,520 (47.51)	37.00 (33.00, 43.00)	22.00 (19.00, 25.00)	16.00 (12.00, 19.00)
5–10	1,120 (35.01)	38.00 (33.00, 44.00)	23.00 (19.00, 26.00)	16.00 (11.00, 19.00)
> 10	559 (17.47)	40.00 (33.00, 46.00)	24.00 (20.50, 27.00)	16.00 (11.00, 19.00)
χ^2^	–	32.79*	62.23*	2.67*
*P*	–	< 0.001	< 0.001	0.263
**Position**				
Clinical	2,940 (91.90)	38.00 (33.00, 44.00)	22.00 (19.00, 25.00)	16.00 (12.00, 19.00)
Administrative	259 (8.10)	40.00 (34.00, 45.00)	24.00 (21.00, 27.00)	15.00 (10.00, 18.00)
Z	–	−3.28	−5.87	−4.44
*P*	–	0.001	< 0.001	< 0.001
**Previous delirium nursing**				
Yes	2,832 (88.53)	38.00 (33.00, 44.00)	23.00 (20.00, 26.00)	16.00 (13.00, 19.00)
No	257 (8.03)	35.00 (31.00, 41.00)	20.00 (18.00, 24.00)	12.00 (10.00, 16.00)
Uncertainty	110 (3.44)	33.00 (29.00, 35.00)	19.00 (18.00, 22.00)	10.50 (9.00, 14.00)
χ^2^	–	96.44*	87.62*	177.30*
*P*	–	< 0.001	< 0.001	< 0.001
**Previous delirium training**				
Yes	2,681 (83.81)	38.00 (33.00, 44.00)	23.00 (19.00, 26.00)	17.00 (13.00, 19.00)
No	518 (16.19)	35.00 (31.00, 39.00)	21.00 (18.00, 24.00)	12.00 (9.00, 15.00)
Z	–	−9.91	−7.82	−17.73
*P*	–	< 0.001	< 0.001	< 0.001

*Kruskal-waills test. ICU, intensive care unit; Z, Mann-Whitney test; M, median; Q1, 1st quartile; Q3, 3st quartile.

### 3.2 Descriptive of KAP scores

**FIGURE 1:**
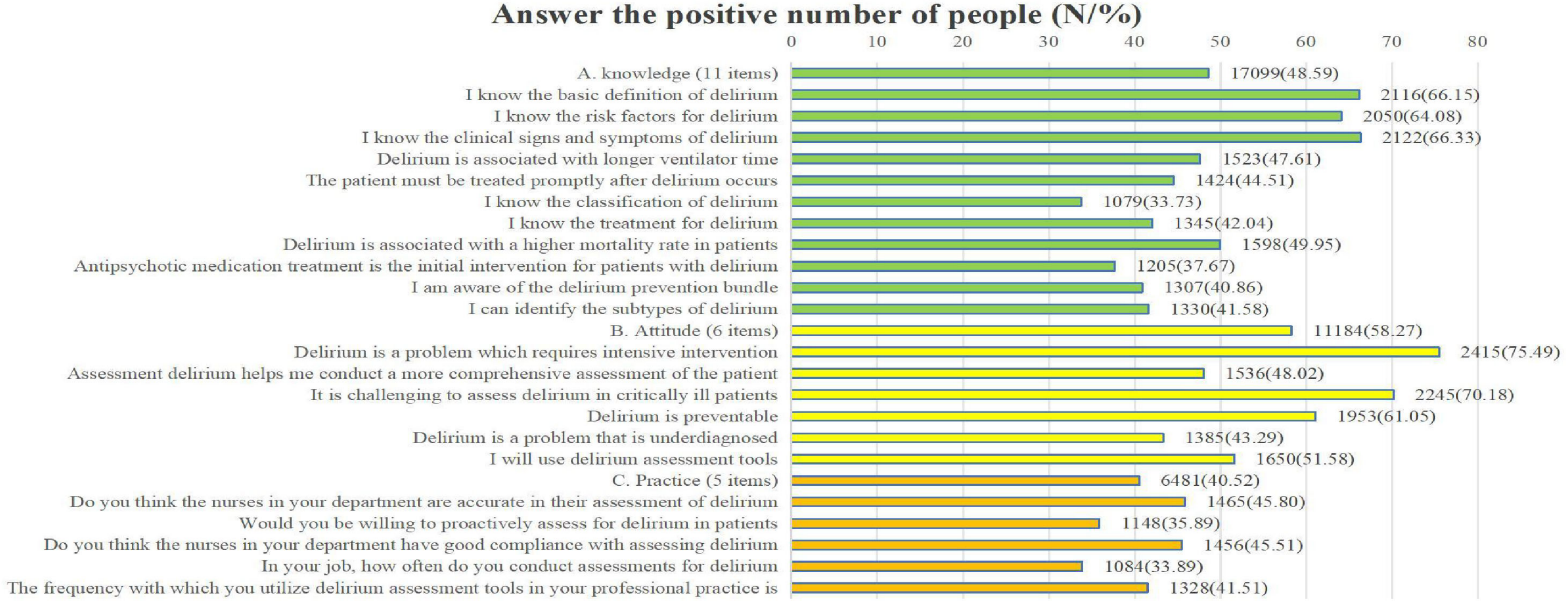
Count of participants reporting positive responses.

As shown in Figure 1, the total score for ICU nurses’ delirium knowledge was 38.00 (33.00, 44.00), with 48.59% of respondents answering positively. The proportion of respondents who correctly identified the types of delirium (33.73%) and recognized antipsychotics as a treatment intervention for delirium (37.37%) was relatively low. The attitude score was 23.00 (19.00, 26.00), with 58.27% of respondents answering positively. Delirium poses a diagnostic challenge (43.29%), with the proportion of individuals responding positively being the lowest among all categories assessed. The overall practice score was 16.00 (12.00, 19.00), with a positive response rate of 40.52%. The number of respondents who frequently conduct delirium assessments (33.89%) and those who actively perform such assessments (35.89%) is relatively low.

### 3.3 Correlations between knowledge, attitude, and practice

This study examines the relationships between knowledge, attitude, and practice scores using the Pearson correlation coefficient. Table 2 reveals a statistically significant link between nurses’ attitude scores and their knowledge levels (*p* < 0.001). Additionally, practice scores showed strong correlations with both knowledge (*p* < 0.001) and attitude scores (*p* < 0.001), indicating meaningful connections among these variables.

**TABLE 2:** Correlation analysis results of knowledge, attitude, and practice.

Project	Knowledge	Attitude	Practice
Knowledge	1	–	–
Attitude	0.774**	1	–
Practice	0.409**	0.252**	1

**At the 0.01 significance level (two-tailed), the correlation is significant.

### 3.4 Multivariate linear regression analysis of nurses’ knowledge, attitude, and practice toward delirium

Based on the Spearman correlation analysis results, we excluded the variables with high correlation (“ICU Work Duration”) in the knowledge dimension [ρ(3197) = 0.735, *p* < 0.001] and the attitude dimension [ρ(3197) = 0.735, *p* < 0.001], and included the remaining variables in the multiple linear regression analysis. As shown in Table 3, the results of the multiple linear regression analysis shows that primary hospitals (β = 0.92, *P* = 0.006) and secondary hospitals (β = 0.71, *P* = 0.04) have significantly higher knowledge scores than tertiary hospitals. Furthermore, private hospitals outperform public ones (β = 2.02, *P* < 0.001). Additionally, those with primary titles showed higher knowledge scores than intermediate titles (β = 1.8, *P* < 0.001). Service years (β = 1.4, *P* = 0.007), delirium care experience (β = 2.53, *P* < 0.001), and delirium training (β = 3.42, *P* < 0.001) are positively correlated with knowledge scores. Attitude scores are higher in first-level (β = 0.52, *P* = 0.004) than in tertiary hospitals, with private hospitals scoring better than state ones (β = 1.38, *P* < 0.001). A bachelor’s degree (β = 0.6, *P* < 0.001), senior title (β = 1.29, *P* = 0.02), experience in delirium care (β = 1.35, *P* < 0.001), and training in delirium (β = 0.27, *P* < 0.001) are positively correlated with knowledge scores. A higher level of education (β = 1.39, *P* = 0.037), surgical ICU (β = 0.66, *P* = 0.02), private hospital (β = 1.17, *P* < 0.001), clinical position (β = 0.87, *P* = 0.007), shorter working hours (β = 0.84, *P* < 0.001), primary professional title (β = 1.27, *P* < 0.001), and experience in delirium care (β = 3.12, *P* < 0.001), and training (β = 3.75, *P* < 0.001)were related to higher practice scores.

**TABLE 3:** Multivariate regression analysis results influencing delirium knowledge, attitude, and practice (KAP) scores.

Knowledge	β	S.E	t	*P*	β (95% CI)
Intercept	37.73	0.28	133.31	< 0.001	37.73 (37.18∼38.29)
**Hospital level**					
Tertiary hospital					0.00 (Reference)
Secondary hospital	0.71	0.35	2.05	0.04	0.71 (0.03∼1.39)
First-level hospital	0.92	0.33	2.77	0.006	0.92 (0.27∼1.56)
**Hospital nature**					
State hospital					0.00 (Reference)
Private hospital	2.02	0.35	5.77	< 0.001	2.02 (1.33∼2.71)
**Professional title**					
Primary					0.00 (Reference)
Intermediate	−1.8	0.48	−3.74	< 0.001	−1.80 (−2.75∼−0.86)
Senior	0.4	1.01	0.39	0.694	0.40 (−1.59∼2.39)
**Service years**					
< 5					0.00 (Reference)
5–10	0.82	0.41	1.98	0.048	0.82 (0.01∼1.63)
> 10	1.4	0.52	2.71	0.007	1.40 (0.39∼2.42)
**Previous delirium nursing**					
Yes					0.00 (Reference)
No	−2.53	0.51	−4.94	< 0.001	−2.53 (−3.54∼−1.53)
Uncertainty	−5.56	0.75	−7.36	< 0.001	−5.56 (−7.03∼−4.08)
**Previous delirium training**					
Yes					0.00 (Reference)
No	−3.42	0.37	−9.18	< 0.001	−3.42 (−4.15∼−2.69)
**Attitude**					
Intercept	21.8	0.28	77.13	< 0.001	21.80 (21.24∼22.35)
**Hospital level**					
Tertiary hospital					0.00 (Reference)
Secondary hospital	0.2	0.19	1.06	0.291	0.20 (−0.17∼0.57)
First-level hospital	0.52	0.18	2.91	0.004	0.52 (0.17∼0.88)
**Hospital nature**					
State hospital					0.00 (Reference)
Private hospital	1.38	0.19	7.09	< 0.001	1.38 (1.00∼1.76)
**Education**					
Associate degree and below					0.00 (Reference)
Bachelor’s degree	0.6	0.16	3.7	< 0.001	0.60 (0.28∼0.91)
Master’s degree and above	0.84	0.7	1.21	0.228	0.84 (−0.53∼2.21)
**Professional title**					
Primary					0.00 (Reference)
Intermediate	−0.38	0.27	−1.41	0.158	−0.38 (−0.90∼0.15)
Senior	1.29	0.56	2.32	0.02	1.29 (0.20∼2.39)
**Previous delirium nursing**					
Yes					0.00 (Reference)
No	−1.35	0.28	−4.84	< 0.001	−1.35 (−1.90∼−0.80)
Uncertainty	−2.4	0.41	−5.86	< 0.001	−2.40 (−3.21∼−1.60)
**Previous delirium training**					
Yes					0.00 (Reference)
No	−1.27	0.2	−6.27	< 0.001	−1.27 (−1.67∼−0.88)
**Practice**					
Intercept	16.36	0.28	58.52	< 0.001	16.36 (15.82∼16.91)
**Hospital nature**					
State hospital					0.00 (Reference)
Private hospital	1.17	0.19	6.29	< 0.001	1.17 (0.81∼1.53)
**Department**					
medical ICU					0.00 (Reference)
surgical ICU	0.66	0.28	2.33	0.02	0.66 (0.11∼1.22)
general ICU	0.11	0.24	0.45	0.649	0.11 (−0.35∼0.57)
**Age(years)**					
< 30					0.00 (Reference)
30–40	0.45	0.15	3.07	0.002	0.45 (0.16∼0.74)
> 40	0.39	0.73	0.54	0.589	0.39 (−1.03∼1.82)
**Education**					
Associate degree and below					0.00 (Reference)
Bachelor’s degree	−0.25	0.15	−1.64	0.101	−0.25 (−0.55∼0.05)
Master’s degree and above	1.39	0.66	2.08	0.037	1.39 (0.08∼2.69)
**Professional title**					
Primary					0.00 (Reference)
Intermediate	−1.27	0.25	−4.98	< 0.001	−1.27 (−1.76∼−0.77)
Senior	0.11	0.53	0.21	0.832	0.11 (−0.92∼1.15)
**Service years**					
< 5					0.00 (Reference)
5–10	−0.59	0.18	−3.33	< 0.001	−0.59 (−0.94∼−0.24)
> 10	−0.84	0.2	−4.09	< 0.001	−0.84 (−1.24∼−0.44)
**Position**					
Clinical					0.00 (Reference)
Administrative	−0.87	0.32	−2.69	0.007	−0.87 (−1.50∼−0.24)
**Previous delirium nursing**					
Yes					0.00 (Reference)
No	−3.12	0.27	−11.78	< 0.001	−3.12 (−3.64∼−2.60)
Uncertainty	−4.11	0.39	−10.51	< 0.001	−4.11 (−4.88∼−3.34)
**Previous delirium training**					
Yes					0.00 (Reference)
No	−3.75	0.19	−19.37	< 0.001	−3.75 (−4.12∼−3.37)

ICU, intensive care unit.

## 4 Discussion

Delirium is a sudden-onset neurological disorder characterized by acute confusion, attention deficits, and fluctuating consciousness (1, 2). Left unmanaged, it can lead to serious complications (30). Nurses play a pivotal role in identifying, managing, and providing care for delirium patients, as their KAP directly impact clinical outcomes. This study utilized a custom-designed questionnaire to evaluate ICU nurses’ KAP concerning delirium. Findings revealed median scores of 38.00 (33.00, 44.00) for knowledge, 23.00 (19.00, 26.00) for attitude, and 16.00 (12.00, 19.00) for practice, with positive response rates of 48.59%, 58.27%, and 40.52%, respectively— consistent with Xing et al.’s (31) research. The data suggests ICU nurses’ KAP levels remain suboptimal, largely because they acquire delirium-related information through scattered online resources rather than structured education programs, ultimately limiting their competency (31).

To our knowledge, this research represents the most extensive multi-center cross-sectional investigation to date examining Chinese ICU nurses’ KAP concerning delirium, while also identifying key contributing factors. The research results show that the level of ICU nurses’ knowledge of delirium is highly consistent with the previous survey results (49.8%) (32). In this study, the correct answer rate was 48.59%, significantly higher than the Ethiopian research data (29.21%) (33), but lower than the result of a certain study in the United States (59%) (34). These discrepancies likely stem from differences in national healthcare policies, economic conditions, participant demographics, professional backgrounds, ongoing training initiatives, assessment methodologies, and sample sizes. Notably, awareness of delirium subtypes and the use of antipsychotics as first-line treatments remained concerningly low, with only 33.73% and 37.37% of respondents answering correctly. Existing literature suggests that hospital-based training programs on delirium diagnosis, prevention, and management guidelines may lack sufficient depth and practical application, warranting further scrutiny (35).

The findings from the multifactorial analysis show that primary, secondary, and private medical institutions outshine their peers when it comes to knowing about delirium. This disparity can be attributed to a few key factors. Firstly, there’s the policy push by lower-tier hospitals to bolster their assessment and treatment skills. Secondly, the high number of elderly patients they care for presents them with real-life experiences to learn from. Large hospitals, on the other hand, get pulled in different directions due to the sheer volume of cases. When it comes to nursing titles, those with more middling ranks have less expertise than their subordinates, mainly because they tend to gravitate toward administrative duties in their units. There’s a clear link between tenure and knowledge of delirium; newer nurses are often found lacking, a trend mirrored by earlier research (36). These earlier studies noted that although there are some neurology topics in the universities’ curricula, delirium is just given a fleeting nod, with no systematic exploration of delirium care. What’s more, delirium isn’t given top billing in nursing education. However, nurses who’ve tended to delirious patients and undergone specific training exhibit a notably higher level of understanding compared to their untrained counterparts. This suggests that current training programs are yielding positive, albeit limited, results. According to Fu et al.’s (37) study, educational interventions successfully improve nurses’ understanding of delirium. Consequently, administrators should strengthen educational reforms to reinforce foundational knowledge, utilize policy measures to address implementation challenges, and integrate clinical support tools to streamline practical application. This approach fosters a self-reinforcing cycle of “knowledge acquisition, improved practice, and enhanced outcomes.” Particular emphasis should be placed on mid-career nurses to ensure they balance managerial duties with ongoing professional development.

The study revealed that 58.27% of participants held a favorable view, a figure that falls short of Sri Lanka’s 80% (38) but surpasses Australia’s 9% (36). These disparities likely stem from regional distinctions, varying degrees of government involvement, and differences in research methodologies and sample sizes. Multivariate analysis suggests that primary and secondary hospitals outperform tertiary facilities in their approach to delirium assessment. Similarly, private hospitals exhibit greater proficiency in evaluating delirium compared to public institutions, possibly due to differences in workload and nurse stress levels. Nurses with bachelor’s degrees demonstrated more positive attitudes toward delirium assessment, likely because of their enhanced learning abilities and clinical reasoning skills (39). Likewise, senior-level nurses showed greater competence in delirium evaluation, owing to their extensive experience, teaching backgrounds, and higher motivation. Notably, nurses who had previously cared for delirium patients or undergone specialized training displayed significantly more favorable attitudes. To improve efficiency, larger hospitals could streamline delirium assessment by incorporating it into routine tasks like vital sign checks or adjusting shift schedules. For new hires, pairing them with experienced mentors for clinical guidance could prove beneficial. Furthermore, implementing incentive programs—such as highlighting successful delirium care cases—could foster greater enthusiasm and professionalism among nursing staff.

Only 40.52% of ICU nurses demonstrated positive delirium management practices, highlighting a gap between knowledge and implementation. This issue may be related to the lack of standardized procedures within the department, improper use of tools, and the failure to incorporate delirium assessments into routine nursing care (40). Compared to medical ICUs, surgical ICU nurses are more proactive in conducting delirium assessments. This may be related to the relatively homogeneous nature of surgical patients’ conditions, which facilitates evaluation. Nurses with higher education are more proactive in assessing and preventing delirium. Their advanced training enhances their focus on these evaluations (41). The study findings reveal an unexpected trend: nurses with senior titles, extensive experience, or leadership roles often demonstrate weaker performance in delirium assessment. This gap appears to stem from limited hands-on practice, which has compromised their evaluation capabilities. To address this, nursing administrators should weave delirium screening into standard care protocols. Hands-on experience with delirious patients and targeted training significantly improves performance across knowledge, attitudes, and practical skills—highlighting the importance of blending theoretical instruction with real-world application. For example, training effectiveness can be boosted through case-based learning and periodic skill evaluations (42, 43). Moving forward, programs should prioritize instruction on symptom recognition, risk assessment, preventive strategies, and care interventions. Nursing leaders would benefit from assembling specialized teams to implement structured training initiatives—such as micro-courses, simulated scenarios, clinical judgment exercises, and case review sessions—to holistically strengthen delirium care competencies.

This study has several limitations. First, the questionnaire used in this research was self-designed; although it underwent expert review, it has not yet been subjected to relevant psychological validation. Additionally, the data collection method—distributing questionnaires via WeChat—may have introduced some bias. There are two primary concerns here: first, the nature of online self-reporting means some participants might have received assistance, potentially skewing response patterns; second, China’s regional economic disparities, particularly between the more developed eastern provinces and the less affluent western areas, could further distort the sample’s representativeness. Another potential limitation stems from the involvement of hospital administrators in relaying research procedures and objectives. This indirect communication channel may have led to inconsistencies in how information was conveyed, possibly influencing participant responses and, ultimately, the study’s outcomes.

## 5 Conclusion

Our research indicates that ICU nurses possess a moderate level of knowledge and exhibit a generally positive attitude toward delirium; however, they demonstrate a relatively passive approach in practice, indicating the importance of improving nurses’ education level, training, and experiential learning related to delirium. Based on comprehensive research findings, we recommend that hospital management develop more specific training programs tailored to factors such as management hierarchy, professional titles, and training experience. It is crucial to emphasize the integration of practical application with theoretical knowledge while also summarizing experiences to improve knowledge, attitudes, and practices related to delirium.

## Data Availability

The raw data supporting the conclusions of this article will be made available by the authors, without undue reservation.
