# Supplementary material for: Knowledge, attitude, and practice of intensive care nurses toward delirium: a multicenter cross-section study
Source: Front Med (Lausanne). 2025 Sep 10;12:1552923. doi: 10.3389/fmed.2025.1552923 (PMC12457401; doi:10.3389/fmed.2025.1552923)
Supplement: Supplementary file 1 [file Data_Sheet_1.pdf]

Supplementary Material

1 Supplementary Figures

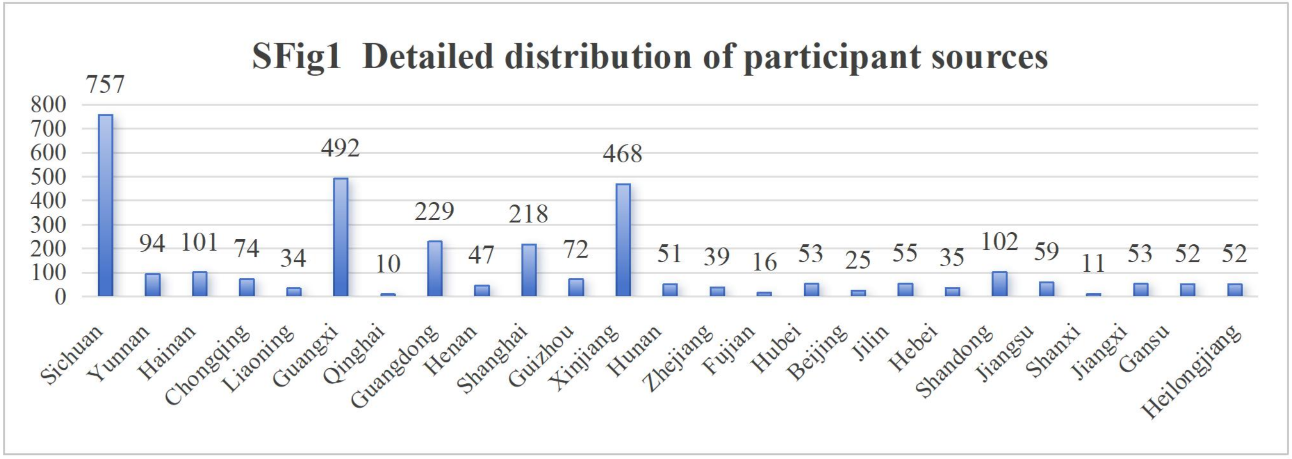

Confirmatory Factor Analysis (CFA) Fit Indices

**Fit Indices**	χ2/df	RMSEA	GFI	AGFI	IFI	TLI	CFI
Result	2.004	0.062	0.868	0.838	0.933	0.924	0.932

Note: χ^2/df：Chi-Square/Degrees of Freedom，CFI：Comparative Fit Index，RMSEA：Root Mean Square Error of Approximation，TLI：Tucker-Lewis Index

Convergent Validity

Latent variable	Observation indicators	Factor Loadings	CR	AVE
Knowledge	K1	0.675	0.919	0.513
	K2	0.735		
	K3	0.684		
	K4	0.579		
	K5	0.807		
	K6	0.787		
	K7	0.612		
	K8	0.647		
	K9	0.787		
	K10	0.649		
	K11	0.856		
Attitude	A1	0.642	0.875	0.542
	A2	0.634		
	A3	0.835		
	A4	0.870		
	A5	0.717		
	A6	0.687		

Practice	P1	0.697	0.847	0.528
	P2	0.722		
	P3	0.628		
	P4	0.833		
	P5	0.739		

Note：CR：Composite Reliability，　AVE：Average Variance Extracted

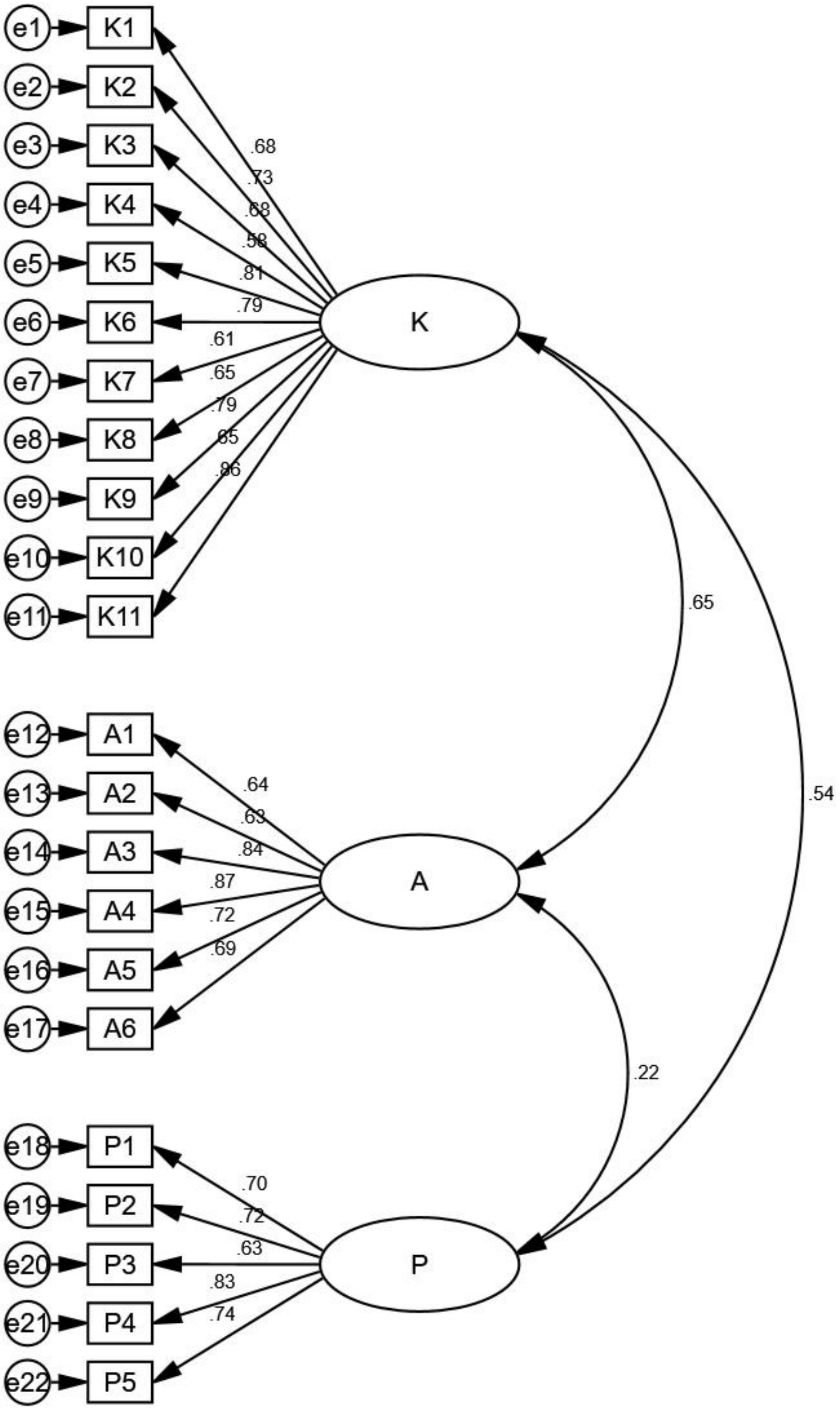
e1
K1
e2
K2
e3
K3
e4
K4
e5
K5
e6
K6
e7
K7
e8
K8
e9
K9
e10
K10
e11
K11
.68
.73
.68
.58
.81
.79
.61
.65
.79
.65
.86
K
.65
.54
e12
A1
e13
A2
e14
A3
e15
A4
e16
A5
e17
A6
.64
.63
.84
.87
.72
.69
A
.22
e18
P1
e19
P2
e20
P3
e21
P4
e22
P5
.70
.72
.63
.83
.74
P
